# Decreasing the steroid rapidly may help to improve the clinical outcomes of patients with intestinal steroid-refractory acute graft-versus-host disease receiving basiliximab treatment

**DOI:** 10.3389/fonc.2024.1390438

**Published:** 2024-03-26

**Authors:** Cong Cheng, Dao-Xing Deng, Xiao-Hui Zhang, Lan-Ping Xu, Yu Wang, Chen-Hua Yan, Huan Chen, Yu-Hong Chen, Wei Han, Feng-Rong Wang, Jing-Zhi Wang, Yu-Qian Sun, Xiao-Jun Huang, Xiao-Dong Mo

**Affiliations:** ^1^ Research Unit of Key Technique for Diagnosis and Treatments of Hematologic Malignancies, Institute of Hematology & Blood Diseases Hospital, Chinese Academy of Medical Sciences & Peking Union Medical College, Beijing, China; ^2^ Peking University People’s Hospital, Peking University Institute of Hematology, National Clinical Research Center for Hematologic Disease, Beijing Key Laboratory of Hematopoietic Stem Cell Transplantation, Beijing, China; ^3^ Peking-Tsinghua Center for Life Sciences, Academy for Advanced Interdisciplinary Studies, Peking University, Beijing, China

**Keywords:** steroid refractory, acute graft-versus-host disease, basiliximab, steroid decrease protocol, hematopoietic stem cell transplantation

## Abstract

Intestinal steroid refractory acute graft-versus-host disease (SR-aGVHD) is the major cause of mortality in allogeneic hematopoietic stem cell transplantation (allo-HSCT). This retrospective cohort study aimed to identify the relationship between different steroid decreasing velocity and therapeutic response in patients with intestinal SR-aGVHD receiving basiliximab treatment, and also aimed to propose a reasonable steroid decreasing regimen for these patients. The median time for steroid dose decreasing to the 50% of initial dose and decreasing to the low-dose steroid for patients achieving ORR was 5 days and 12 days, respectively, which was both shorter than patients without achieving ORR. The ORR, NRM and survival in rapid and medium steroid decreasing group were all better than slow group. The cumulative incidence of ORR at any time was 90.4%, 78.1% and 62.3%, respectively, in rapid, medium, and slow group. The cumulative incidence of NRM at 1 year after basiliximab treatment was 18.7% (95% CI 11.3%–26.1%), 22.8% (95% CI 14.2%–31.4%) and 32.8% (95% CI 24.1%–41.5%), respectively, in rapid, medium, and slow group. The probability of OS at 1 year after basiliximab treatment was 76.9% (95% CI 68.9%–84.9%), 72.7% (95% CI 63.7%–81.7%), and 62.3% (95% CI 53.5%–71.1%), respectively, in rapid, medium, and slow group. Hence, it was helpful to decrease steroid to the 50% of initial dose ≤ 5 days and to the low-dose steroid ≤ 12 days after basiliximab treatment for intestinal SR-aGVHD patients, which may also be the reasonable steroid decrease protocol for these patients.

## 1 Introduction

Allogeneic hematopoietic stem cell transplantation (allo-HSCT) is effective treatment for hematologic malignancies (1–5). Acute graft-versus-host disease (aGVHD) remains an important complication and one of the leading causes of non-relapse mortality (NRM) after allo-HSCT (6–8). More than half of GVHD patients showed gastrointestinal tract involvements (9), and patients with severe intestinal aGVHD have a high risk of GVHD-associated mortality. A study enrolled 1462 allo-HSCT recipients showed that although the incidence of grade III-IV intestinal aGVHD was only 7.9%, the 2-year probability of overall survival (OS) rate of these patients was as low as 25% (10).

Although corticosteroids remain the first-line treatment for aGVHD (11, 12), only 30-50% of patients showed response to corticosteroid therapy (13, 14) and a significant portion of these patients would experience steroid-refractory aGVHD (SR-aGVHD) (15). The clinical outcomes of SR-aGVHD patients were poor and the mortality rate could be as high as 90% (16).

Basiliximab, a chimeric monoclonal antibody binding to the α-chain of interleukin 2 receptor (IL-2R) with high affinity, is an effective treatment for SR-aGVHD (17–19). Our previous study found that for the SR-aGVHD, overall response rate (ORR) at day 28 after basiliximab treatment was 65.7%-79.4%, which was 67.3% for those with intestinal SR-aGVHD (18, 20). However, these studies did not compare the cumulative dose of steroid between patients with and without ORR after basiliximab treatment and whether basiliximab treatment could indeed decrease the dose of steroid was unclear.

In addition, how to decrease the steroid in SR-aGVHD patients receiving second-line treatment was controversial. Some physicians suggested that second-line treatment may have synergistic effect with steroid and decreasing the steroid rapidly may further deteriorate the situation of SR-aGVHD. However, continually using high-dose steroids may lead to serious complications, such as increasing the risk of infection (21, 22), disturbing gut microbiota (23), inducing cutaneous atrophy (24, 25) and delaying wound healing (26–28). It could also cause other adverse effects of gastrointestinal (29, 30), such as gastric ulcer (31), colon perforation (32), and gastrointestinal bleeding (33–35). Therefore, decreasing the steroid properly may help to control the severe intestinal SR-GVHD, but the reasonable protocol of steroid decreasing in these patients was still unclear.

In this retrospective study, we aimed to identify the relationship between different steroid decreasing velocity and therapeutic response in patients with intestinal SR-aGVHD who received basiliximab treatment. We also aimed to propose a reasonable steroid decrease regimen for these patients.

## 2 Methods

### 2.1 Patients

During the period from January 1, 2015 to December 31, 2018, patients diagnosed as intestinal SR-aGVHD and received basiliximab treatment after allo-HSCT in the Institute of Hematology, Peking University (PUIH) were enrolled in this study. The major clinical outcomes of these patients had been reported previously as parts of a multicenter real-world study (18), and these patients were further followed in this study. The study was carried out in accordance with the Declaration of Helsinki. This study protocol was approved by the Ethics Committee of Peking University People’s Hospital.

### 2.2 Treatment of aGVHD

Methylprednisolone (MP) was the first-line treatment for newly diagnosed aGVHD. 2 mg·kg^−1^·day^−1^ of MP for patients aged < 10 years or weighing < 30 kg, and the others received MP at a dose of 1 mg·kg^−1^·day^−1^. If the patient was diagnosed with SR-aGVHD (36), treatment with basiliximab was initiated. Recommended dose of basiliximab was as followed: 20 mg per dose for adults and children weighing ≥ 35 kg, or 10 mg per dose for children weighing < 35 kg. Basiliximab was administered on +1, +3, +8 days, and once a week thereafter until the aGVHD was less than grade II (18).

### 2.3 Definition

Complete response (CR) referred to the complete disappearance of all signs and symptoms of aGVHD in all assessable organs without additional systemic treatment. Partial response (PR) was defined as one stage improvement in one or more organs involved in the signs or symptoms of aGVHD, without progression in other organs and without additional systemic treatment. The overall response rate (ORR) was defined as CR plus PR.

Definition of the low-dose steroid criteria: steroid decreasing to ≤ 0.2 mg/kg in patients < 40 kg of body weight, or ≤ 10 mg/day in patients of > 40 kg of body weight (37).

NRM referred to all deaths without underlying disease recurrence. OS was defined as the time from treatment with basiliximab to death from any cause. Disease free survival (DFS) was defined as the survival duration of continuous CR after basiliximab treatment.

aGVHD grade was based on the Mount Sinai Acute GVHD International Consortium (MAGIC) and Minnesota Acute GVHD Risk Score (36, 38). Hematopoietic Cell Transplantation-Specific Comorbidity Index (HCT-CI) scores were reported according to the results of Sorror et al. (39).

### 2.4 Statistical analysis

For the comparisons of patient characteristics, categorical variables were calculated using the chi-squared test or Fisher’s exact test. Continuous variables were analyzed using t-tests or Mann–Whitney U tests. The probability of survival was analyzed using the Kaplan–Meier method. The cumulative incidence of ORR, NRM, and GVHD were estimated using competing risk regression analysis.

Hazard ratios (HRs) for steroid decreasing velocity and other clinical outcomes were estimated with ORR, NRM, OS, and DFS in a multivariable analysis using Cox proportional hazards regression with a backward stepwise model selection approach. Independent variables with *P* values >.1 were sequentially excluded from the model, and *P* values <.05 (two-tailed) was considered statistically significant. Statistical analyses were performed using SPSS 26 (SPSS Inc./IBM, Armonk, NY, USA), the R software package (version 4.3.0; http://www.r-project.org) and Prism 9 (GraphPad Software).

## 3 Results

### 3.1 Patient characteristics

A total of 314 patients with intestinal SR-aGVHD were enrolled (**Table 1**). The median time from diagnosis of intestinal aGVHD to basiliximab treatment was 5 (range, 3 to 23) days. The median doses of basiliximab were 4 (range, 2 to 14) doses. The median time from the beginning of basiliximab treatment to achieving ORR and CR was 6 (range, 2 to 65) days and 10 (range, 3 to 95) days, respectively. The cumulative incidence of ORR and CR at day 28 after basiliximab treatment was 70.1% and 58.0%, respectively. The cumulative incidence of ORR and CR at any time after basiliximab treatment was 76.4% and 65.3%, respectively. The probability of OS, DFS, and NRM at 3 years after basiliximab treatment was 66.9% (95% CI 61.6%-71.5%), 64.4% (95% CI 59.1%-69.7%), and 25.9% (95% CI 21.1%-30.9%), respectively.

**Table 1:** Patient characteristics.

Variable	*n*=314
Age at HSCT, *n* (%)	
Mean age, y (SD)	27.4 (15)
< 18 years	93 (10)
≥ 18 years	221 (34)
Female sex, *n* (%)	139 (44.3)
Underlying disease, *n* (%)	
Hematologic malignancies	266 (84.7)
Acute leukemia	200 (63.7)
Myelodysplastic syndrome	44 (14.0)
chronic myelogenous leukemia	7 (2.2)
Myeloproliterative neoplasms	6 (1.9)
Malignant lymphoma	6 (1.9)
Multiple myeloma	1 (0.3)
Others	2 (0.6)
Nonmalignant hematologic disease	47 (15.0)
Severe aplastic anemia	42 (13.4)
Others	5 (1.6)
HCT-CI score, *n* (%)	
Low risk	226 (72.0)
Intermediate risk	67 (21.3)
High risk	21 (6.7)
Donor–recipient relationship, *n* (%)	
Father–child	160 (51.0)
Mother–child	12 (3.8)
Sibling–sibling	87 (27.7)
Child–parent	44 (14.0)
Collateral related donor	6 (1.9)
Unrelated donor	5 (1.6)
Donor-recipient sex matched, *n* (%)	
Male to male	133 (42.4)
Male to female	105 (33.4)
Female to male	42 (13.4)
Female to female	34 (10.8)
Donor type, *n* (%)	
Identical sibling donor	31 (9.9)
Haploidentical donor	278 (88.5)
Unrelated donor	5 (1.6)
Cytomegalovirus serology, *n* (%)	
Recipient negative, donor negative	14 (1.5)
Recipient negative, donor positive	13 (1.4)
Recipient positive, donor negative	26 (2.8)
Recipient positive, donor positive	739 (78.6)
Not available	148 (15.7)
Graft type, *n* (%)	
Bone marrow and peripheral blood stem cells	297 (94.6)
Peripheral blood stem cells	17 (5.4)
Conditioning regimen, *n* (%)	
Chemotherapy based regimen	305 (97.1)
Total body irradiation based regimen	9 (2.9)
Platelet engraftment, *n* (%)	277 (88.2)
Mean time from HSCT to platelet engraftment, d (SD)	66 (173)
Mean follow-up after basiliximab treatment, d (SD)	805 (566)

### 3.2 Steroid decreasing velocity after basiliximab and therapeutic response

The median time from the beginning of basiliximab treatment to steroid dose decreased to the 50% of initial dose was 6 (range, 1 to 21) days, and the median time from the beginning of basiliximab treatment to steroid dose decreased to the low-dose steroid was 13 days (range, 2 to 45) days. The median time for steroid dose decreased to the 50% of initial dose and decreased to the low-dose steroid was 5 (range, 1-16) days versus 7.5 (range, 2-21) days (*P* < 0.001) and 12 (range, 2-33) days versus 15 (range, 5-45) days (*P* = 0.003), respectively, for patient with and without achieving ORR at day 28. The median time for steroid dose decreased to the 50% of initial dose and decreased to the low-dose steroid was 5 (range, 1-21) days versus 7.5 (range, 3-16) days (*P* < 0.001) and 12 (range, 2-44) days versus 15 (range, 5-45) days (*P* = 0.008), respectively, for patient with and without achieving ORR at any time. This suggested that patients who achieved ORR or CR showed more rapid steroid decreasing velocity.

Furthermore, for patient with and without achieving ORR at day 28, the median cumulative dose of steroids at 28 days was 8.4 (range, 1.7-26.6) mg/kg versus 10.1 (range, 1.9-44.8) mg/kg (*P* = 0.003). For patient with and without achieving ORR at any time, the median cumulative dose of steroids at any time was 8.5 (range, 1.7-37.9) mg/kg versus 10.1 (range, 1.9-44.8) mg/kg (*P* = 0.006). Meanwhile, the steroid decreasing velocity curves within 28 days in patients achieving ORR at day 28 and at any time were both more rapid than non-ORR patients (**Supplementary Figure 1A**, *P* < 0.001; **Supplementary Figure 1B**, *P* < 0.001). These results suggests that patients achieving ORR after basiliximab treatment required fewer steroids dose than non-ORR patients.

**Figure 1:**
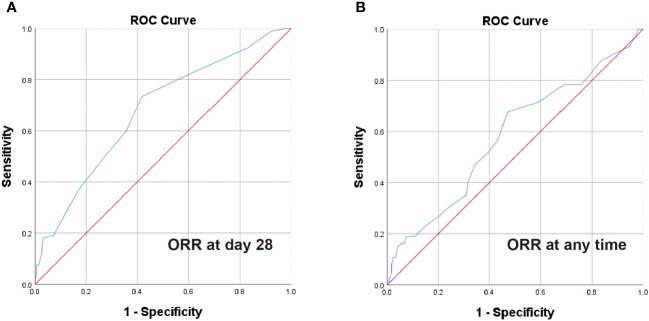
ROC analysis of steroid decrease time for ORR at day 28 and any time. The optimal cut-off point for steroid dose halving time **(A)** was 5 days according to the ORR at day 28 (AUC = 0.677, 95% CI 0.613-0.742, P < 0.001) and steroid dose reduction to low-dose steroid time **(B)** was 12 days according to the ORR at any time (AUC = 0.582, 95% CI 0.506-0.658, P = 0.033).

ROC curve analysis was performed to estimate the optimal cut-off point of steroid decreasing velocity. The optimal cut-off time of steroid dose decreased to the 50% of initial dose of steroid was 5 days according to the ORR at day 28 (**Figure 1A**), and the optimal cut-off time for decreasing steroid dose to the low-dose steroid was 12 days according to the ORR at any time (**Figure 1B**).

**Figure 2:**
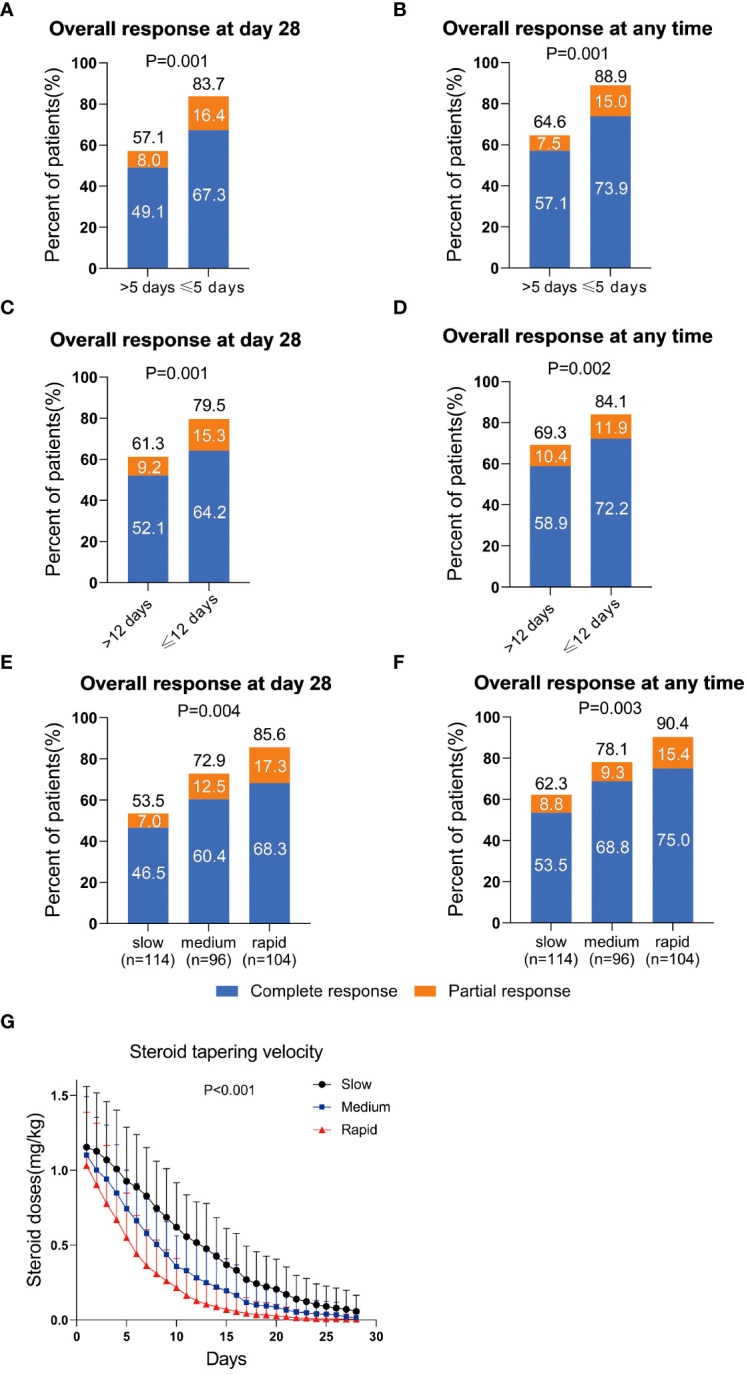
Overall response rate of steroid-refractory acute graft-versus-host disease (SR-aGVHD) patients after basiliximab treatment between steroid decreasing velocity subgroups. **(A)** At day 28, according to the optimal cut-off point for steroid dose halving time; **(B)** at any time, according to the optimal cut-off point for steroid dose halving time; **(C)** At day 28, according to the optimal cut-off point for steroid dose reduction to the low-dose steroid time; **(D)** at any time, according to the optimal cut-off point for steroid dose reduction to the low-dose steroid time; **(E)** At day 28, and **(F)** at any time, integrating between steroid decreasing velocity subgroups; The steroid decreasing velocity curve within 28 days in rapid, medium, and slow group **(G)**.

The ORR at day 28 and at any time of steroid dose halving ≤ 5 days group was 83.7% and 88.9%, respectively, which was both significantly higher than those of halving > 5 days group (at day 28: 57.1%, *P* = 0.001, at any time: 64.6%, *P* = 0.001, **Figures 2A, B**). The ORR at day 28 and at any time of steroid dose decreased to the low-dose steroid ≤ 12 days was 79.5% and 84.1%, respectively, which was also both significantly higher than those of steroid dose decreased to the low-dose steroid >12 days (at day 28: 61.3%, *P* = 0.001, at any time: 69.3%, *P* = 0.002, **Figures 2C, D**). In addition, the steroid dose halving ≤ 5 days group and steroid dose decreased to the low-dose steroid ≤ 12 days group were also showed better CR rates (**Figures 2A–D**).

Thus, the intestinal SR-aGVHD patients were divided into three groups: rapid (steroid dose decreased to the 50% of initial dose ≤ 5 days and decreased to the low-dose steroid ≤ 12 days), medium (steroid dose decreased to the 50% of initial dose ≤ 5 days or decreased to the low-dose steroid ≤ 12 days) and slow (steroid dose decreased to the 50% of initial dose > 5 days and decreased to the low-dose steroid >12 days) group. The steroid decreasing velocity curve within 28 days in rapid, medium, and slow group were shown in **Figure 2G** (*P* < 0.001).

The cumulative incidence of ORR at day 28 was 85.6%, 72.9% and 53.5%, respectively, in rapid, medium, and slow group (*P* = 0.004, **Figure 2E**). The cumulative incidence of ORR at any time was 90.4%, 78.1% and 62.3%, respectively, in rapid, medium, and slow group (*P* = 0.003, **Figure 2F**).

The cumulative incidence of CR at day 28 was 68.3%, 60.4% and 46.5%, respectively, in rapid, medium, and slow group (*P* = 0.008, **Figure 2E**). The cumulative incidence of CR at any time was 75.0%, 68.8% and 53.5%, respectively, in rapid, medium, and slow group (*P* = 0.003, **Figure 2F**).

### 3.3 Steroid decreasing velocity and therapeutic response according to SR-aGVHD severity

In patients with grade II intestinal SR-aGVHD at the beginning of basiliximab treatment, the cumulative incidence of ORR at day 28 after basiliximab treatment was 86.1%, 82.3% and 62.2%, respectively, in rapid, medium, and slow group (*P* = 0.002, **Supplementary Figure 2A**). The cumulative incidence of CR at day 28 after basiliximab treatment was 76.4%, 74.2% and 56.8%, respectively, in rapid, medium, and slow group (*P* = 0.021, **Supplementary Figure 2A**). The cumulative incidence of ORR at any time after basiliximab treatment was 90.3%, 85.5% and 66.2%, respectively, in rapid, medium, and slow group (*P* = 0.001, **Supplementary Figure 2B**). The cumulative incidence of CR at any time after basiliximab treatment was 80.6%, 79.0% and 60.8%, respectively, in rapid, medium, and slow group (*P* = 0.012, **Supplementary Figure 2B**).

In patients with grade III–IV intestinal aGVHD at the beginning of basiliximab treatment, the cumulative incidence of ORR at day 28 after basiliximab treatment was 84.4%, 55.9% and 37.5%, respectively, in rapid, medium, and slow group (*P* = 0.001, **Supplementary Figure 2C**). The cumulative incidence of CR at day 28 after basiliximab treatment was 50.0%, 35.3% and 27.5%, respectively, in rapid, medium, and slow group (*P* = 0.141, **Supplementary Figure 2C**). The cumulative incidence of ORR at any time after basiliximab treatment was 90.6%, 64.7% and 55.0%, respectively, in rapid, medium, and slow group (*P* = 0.004, **Supplementary Figure 2D**). The cumulative incidence of CR at any time after basiliximab treatment was 62.5%, 50.0% and 40.0%, respectively, in rapid, medium, and slow group (*P* = 0.165, **Supplementary Figure 2D**).

### 3.4 Steroid decreasing velocity and new-onset infection after basiliximab treatment

The results of new-onset infections after basiliximab treatment were showed in **Supplementary Table 1**. The rates of new-onset viral infections after basiliximab treatment were 48.1%, 42.7% and 53.5%, respectively, in rapid, medium, and slow group (*P* = 0.295). The rates of new-onset bacterial infections after basiliximab treatment were 10.6%, 16.7% and 14.9%, respectively, in rapid, medium, and slow group (*P* = 0.437). The rates of new-onset fungal infections after basiliximab treatment were 5.8%, 4.2% and 0.9%, respectively, in rapid, medium, and slow group (*P* = 0.141). The rates of any infection (≥ 1 type) were 57.7%, 50.0% and 57.9%, respectively, in rapid, medium, and slow group (*P* = 0.440), and the rates of multiple infections (≥ 2 types) were 13.5%, 18.8% and 19.3%, respectively, in rapid, medium, and slow group (*P* = 0.464).

### 3.5 Steroid decreasing velocity and NRM after basiliximab treatment

A total of 81 patients died from NRM. The most common cause of NRM was infection (45.7%), followed by GVHD (23.5%). The cumulative incidence of NRM at 1 year after basiliximab treatment was 18.7% (95% CI 11.3%–26.1%), 22.8% (95% CI 14.2%–31.4%) and 32.8% (95% CI 24.1%–41.5%), respectively, in rapid, medium, and slow group (*P* = 0.035, **Supplementary Figure 3A**). The cumulative incidence of GVHD-related mortality at 1 year after basiliximab treatment was 0.9% (95% CI 0.2%–1.7%), 6.4% (95% CI 1.4%–11.5%) and 9.7% (95% CI 4.2%–15.2%), respectively, in rapid, medium, and slow group (*P* = 0.011, **Supplementary Figure 3B**). The cumulative incidence of infection-related mortality at 1 years of basiliximab treatment was 9.6% (95% CI 3.9%–15.3%), 11.7% (95% CI 5.2%–18.3%) and 12.3% (95% CI 6.2%–18.4%), respectively, in rapid, medium, and slow group (*P* = 0.692, **Supplementary Figure 3C**).

### 3.6 Steroid decreasing velocity and OS, DFS after basiliximab treatment

**Figure 3:**
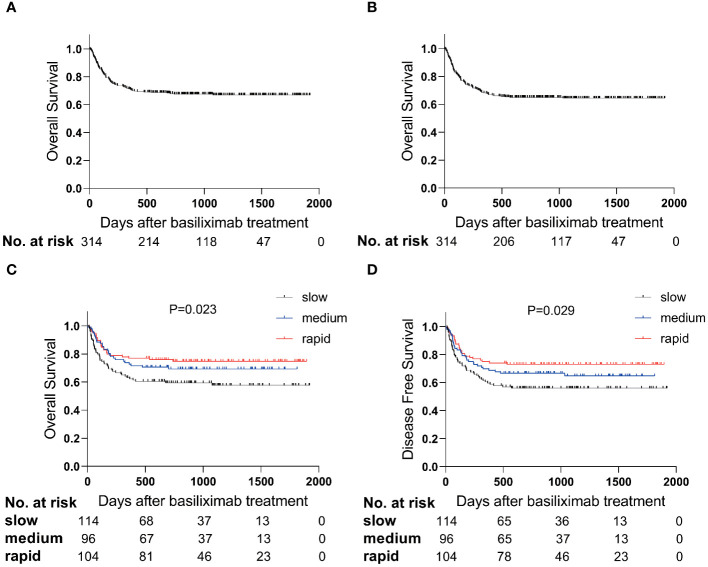
The survival curve after basiliximab treatment. The OS **(A)** and DFS **(B)** in total population; The OS **(C)** and DFS **(D)** between steroid decreasing velocity subgroups.

A total of 212 intestinal SR-aGVHD patients survived after basiliximab treatment, with a median time of OS was 804 (range, 4-1920) days (**Figure 3A**). The probability of OS at 1 year after basiliximab treatment was 76.9% (95% CI 68.9%–84.9%), 72.7% (95% CI 63.7%–81.7%), and 62.3% (95% CI 53.5%–71.1%), respectively, in rapid, medium, and slow group (*P* = 0.024, **Figure 3C**).

The median time of DFS was 777 (range, 4-1920) days (**Figure 3B**). The probability of DFS at 1 year after basiliximab treatment was 75.0% (95% CI 66.8%–83.2%), 69.8% (95% CI 60.6%–79.0%) and 59.6% (95% CI 50.6%–68.6%), respectively, in rapid, medium, and slow group (*P* = 0.029, **Figure 3D**).

### 3.7 The multivariable analysis

In multivariable analysis, grade III–IV aGVHD at the beginning of basiliximab treatment was the risk factor for having a decreased likelihood of response at day 28 and at any time, while rapid steroid decreasing velocity was associated with a higher response at day 28 and at any time (**Supplementary Table 2**). In addition, pretransplant chemotherapy, high-risk refined Minnesota aGVHD risk score and HCT-CI score at the beginning of basiliximab treatment were all the risk factors of OS and DFS (**Supplementary Table 3**). Meanwhile, pretransplant chemotherapy and initial steroid dose ≥ 1mg/kg at the beginning of basiliximab treatment were the risk factors of NRM (**Supplementary Table 3**). Simultaneously, the rapid group (i.e., the SR-aGVHD patients reducing steroid dose to the 50% of initial dose ≤ 5 days and to the low-dose steroid ≤ 12 days after basiliximab treatment) was a favorable prognostic factor for OS, DFS, and decreased likelihood of NRM (**Supplementary Table 3**).

## 4 Discussion

In the present study, we showed that the median time for steroid dose decreasing to the 50% of initial dose and decreasing to the low-dose steroid for patients achieving ORR was 5 days and 12 days, respectively, which were both shorter than patients without achieving ORR. Our results showed that the ORR, NRM, and survival in rapid and medium group were better than those of slow group. Hence, it was reasonable to decrease steroid to the 50% of initial dose ≤ 5 days and to the low-dose steroid ≤ 12 days after basiliximab treatment for intestinal SR-aGVHD patients. Thus far, it was the first study to propose the steroid decrease protocol for these patients.

We observed that the median cumulative dose of steroids at 28 days in patients achieving ORR was 8.5 mg/kg, which was significantly lower than the 10.1 mg/kg of non-ORR patients. It was suggested that effective treatment with basiliximab could indeed decrease the cumulative steroids dose. Similarly, several studies reported that other second-line treatments could also decrease cumulative dose of steroids. Khandelwal et al. (40) found that the majority of SR-aGVHD patients who experienced CR or PR after first alemtuzumab dose were able to wean off high-dose steroids successfully. Reschke et al. (41) reported that compared to non-response patients with steroid-refractory intestinal aGVHD, steroid usage was significantly decreased in patients with PR during extracorporeal photopheresis treatment.

For patient achieving ORR, we found that the median time of steroid dose halving was 5 days, which was earlier than the median time from the beginning of basiliximab treatment to ORR (i.e., 6 days). This result suggested that a significant proportion of patients began steroid decreasing before they achieving ORR after basiliximab treatment, indicating that the decreasing steroid rapidly could help to control intestinal SR-aGVHD. We speculated some reasons may contribute to this result. Firstly, long-term high-dose steroids might cause delayed wound healing in intestinal mucosal injury (26, 42, 43) by inhibiting the proliferation and restitution of intestinal epithelial cells (44–47). Secondly, high-dose corticosteroid therapy could weaken mucosal barrier function (47, 48), change intestinal permeability (49) and intestinal epithelial cell tight junctions (50), which might induce adverse reactions such as its desensitization to sepsis (47, 51, 52) and excessive rectal blood loss (33–35). Lastly, high-dose corticosteroid therapy increased the risk of another adverse effects (29, 30), such as gastric ulcer (31), colon perforation (32), and gastrointestinal bleeding (33–35).

It has been demonstrated that receiving long-term high-dose steroid therapy was an important risk factor for infection (21, 22). However, in our study, the infection rates seemed to be similar in rapid, medium, and slow steroid decreasing groups. The following reasons might contribute to this result. Firstly, GVHD per se could cause an increased risk of infection (53, 54), so even in rapid steroid decrease group, it could not completely eliminate the risk of infection. Secondly, the occurrence of certain infections, such as fungal infections, was relatively low and it was difficult to achieve statistical significance among these three groups. However, we still found that the rates of viral infections and multiple infections in the slow group seems to be higher than those in rapid and medium group, which suggested that decreasing steroid rapidly could help to prevent infection.

The present study has some limitations. Firstly, the relatively small sample size might lead to bias in this retrospective study. Secondly, although the median time of steroid dose halving in patients achieving ORR was earlier than the median time from the beginning of basiliximab treatment to ORR, it could not completely rule out the fact that some patients decrease steroid after they achieving ORR rapidly. Thirdly, at the beginning of this study, ruxolitinib was not widely used as a second-line treatment for SR-aGVHD in China, so we were unable to consider the impact of ruxolitinib on this study. In the future, we can further explore the relationship between different steroid decreasing velocity and therapeutic response in patients with intestinal SR-aGVHD who received ruxolitinib treatment. Future studies on mechanisms *in vivo* and *in vitro* will help further elucidate whether intestinal SR-aGVHD patients receiving basiliximab therapy could benefit from rapid steroid decreasing.

In summary, our results firstly described that rapid steroid decreasing may help to control intestinal SR-aGVHD in patients receiving basiliximab treatment, and we also provide a possible steroid decreasing regimen for these patients. Randomized controlled trials will further confirm our results in future.

## Data availability statement

The raw data supporting the conclusions of this article will be made available by the authors, without undue reservation.

## Ethics statement

The studies involving humans were approved by The Ethics Committee of Peking University People’s Hospital. The studies were conducted in accordance with the local legislation and institutional requirements. The participants provided their written informed consent to participate in this study.

## Author contributions

CC: Data curation, Software, Writing – original draft, Writing – review & editing, Methodology, Visualization. D-XD: Methodology, Writing – review & editing, Software. X-HZ: Data curation, Resources, Writing – review & editing. L-PX: Data curation, Resources, Writing – review & editing. YW: Data curation, Resources, Writing – review & editing. C-HY: Data curation, Resources, Writing – review & editing. HC: Data curation, Resources, Writing – review & editing. Y-HC: Data curation, Resources, Writing – review & editing. WH: Data curation, Resources, Writing – review & editing. F-RW: Data curation, Resources, Writing – review & editing. J-ZW: Data curation, Resources, Writing – review & editing. Y-QS: Data curation, Resources, Writing – review & editing. X-JH: Data curation, Resources, Writing – review & editing, Methodology. X-DM: Data curation, Methodology, Resources, Supervision, Writing – original draft, Writing – review & editing.

## Supplementary material

Supplementary Table 1 New onset infections after basiliximab treatment.

Supplementary Table 2 Multivariate analysis for overall response at day 28 or any time after basiliximab treatment (n=314). GVHD, acute graft-versus-host disease; CI, confidence interval; HR, hazard ratio.

Supplementary Table 3 Multivariate analysis for clinical outcomes after basiliximab treatment (n=314).

Supplementary Figure 1 The steroid decreasing velocity curve within 28 days in patients with or without achieving ORR at any time **(A)** or at day 28 **(B)**.

Supplementary Figure 2 Overall response rate after basiliximab treatment between steroid decrease time subgroups of SR-aGVHD patients divided into grade II aGVHD patients and grade III-IV aGVHD patients according to refined Minnesota aGVHD risk score. The overall response rate **(A)** at day 28 or **(B)** at any time in grade II aGVHD patients; **(C)** at day 28 or **(D)** at any time in grade III-IV aGVHD patients.

Supplementary Figure 3 The cumulative incidence of NRM **(A)**, GVHD related deaths **(B)** and infection related deaths **(C)** after 1 years of basiliximab treatment between steroid decrease time subgroups.
