# Supplementary material for: Decreasing the steroid rapidly may help to improve the clinical outcomes of patients with intestinal steroid-refractory acute graft-versus-host disease receiving basiliximab treatment
Source: Front Oncol. 2024 Mar 26;14:1390438. doi: 10.3389/fonc.2024.1390438 (PMC11002247; doi:10.3389/fonc.2024.1390438)
Supplement: Supplementary Table 1 — New onset infections after basiliximab treatment. [file DataSheet_1.docx]

Supplementary Material

1. **Supplemental tables**

**Supplemental Table 1. New onset infections after basiliximab treatment**

| **Types of infection** | **Slow**  **(n=114)** | **Medium**  **(n=97)** | **Rapid**  **(n=103)** |
| --- | --- | --- | --- |
| **Viral infection** | 61 (53.5%) | 41 (42.7%) | 50 (48.1%) |
| Cytomegalovirus infection | 44 (38.6%) | 29 (30.2%) | 40 (38.5%) |
| Cytomegalovirus DNAemia | 43 (37.7%) | 28 (29.2%) | 35 (33.7%) |
| Cytomegalovirus disease | 4 (3.5%) | 3 (3.1%) | 5 (4.8%) |
| Epstein-Barr virus infection | 19 (16.7%) | 11 (11.5%) | 10 (9.6%) |
| **Bacterial infection** | 17 (14.9%) | 16 (16.7%) | 11 (10.6%) |
| Pneumonia | 13 (11.4%) | 12 (12.5%) | 5 (4.8%) |
| **Fungal infection** | 1(0.9%) | 4(4.2%) | 6(5.8%) |
| **Any infection (≥ 1 type)** | 66 (57.9%) | 48 (50.0%) | 60 (57.7%) |
| **Multiple infection (≥ 2 types)** | 22 (19.3%) | 18 (18.8%) | 14 (13.5%) |

**Supplemental Table 2. Multivariate analysis for overall response at day 28 or any time after basiliximab treatment (n=314)**

| **Variable** | **HR (95% CI)** | ***P*** |
| --- | --- | --- |
| **ORR at day 28** |  |  |
| Severity of aGVHD at the beginning of basiliximab treatment |  |  |
| Grade II | 1 |  |
| Grade III to IV | 0.51 (0.32–0.80) | 0.004 |
| Grouping based on the optimal cut-off point of steroid decrease velocity |  |  |
| Slow | 1 |  |
| Medium | 2.15 (1.43-3.22) | <0.001 |
| Rapid | 3.11 (1.73-5.57) | <0.001 |
| **CR at day 28** |  |  |
| Severity of aGVHD at the beginning of basiliximab treatment |  |  |
| Grade II | 1 |  |
| Grade III to IV | 0.27 (0.14–0.53) | <0.001 |
| Grouping based on the optimal cut-off point of steroid decrease velocity |  |  |
| Slow | 1 |  |
| Medium | 1.99 (1.26-3.14) | 0.003 |
| Rapid | 2.30 (1.18-4.42) | 0.012 |
| **ORR at any time** |  |  |
| Severity of aGVHD at the beginning of basiliximab treatment |  |  |
| Grade II | 1 |  |
| Grade III to IV | 0.55 (0.36–0.83) | 0.004 |
| Grouping based on the optimal cut-off point of steroid decrease velocity |  |  |
| Slow | 1 |  |
| Medium | 2.08 (1.42-3.07) | <0.001 |
| Rapid | 3.13 (1.78-5.47) | <0.001 |
| **Decreased likelihood of CR at any time** |  |  |
| Severity of aGVHD at the beginning of basiliximab treatment |  |  |
| Grade II | 1 |  |
| Grade III to IV | 0.39 (0.23–0.66) | <0.001 |
| Grouping based on the optimal cut-off point of steroid decrease velocity |  |  |
| Slow | 1 |  |
| Medium | 1.89 (1.23-2.89) | 0.004 |
| Rapid | 2.06 (1.12-3.77) | 0.019 |

aGVHD, acute graft-versus-host disease; CI, confidence interval; HR, hazard ratio.

**Supplemental Table 3. Multivariate analysis for clinical outcomes after basiliximab treatment (n=314)**

| **Outcome** | **HR (95% CI)** | | ***P*** | |
| --- | --- | --- | --- | --- |
| **Treatment failure as defined by OS** |  | |  | |
| Refined Minnesota aGVHD risk score before basiliximab treatment |  | |  | |
| Standard risk | 1 | |  | |
| High risk | 1.91 (1.16–3.12) | | 0.010 | |
| HCT-CI score^*^ |  | |  | |
| Low risk | 1 | |  | |
| Intermediate risk | 1.68 (1.04–2.71) | | 0.033 | |
| High risk | 4.52 (2.44–8.37) | | <0.001 | |
| Pretransplant chemotherapy |  | |  | |
| no | 1 | |  | |
| yes | 1.91 (1.24–2.92) | | 0.003 | |
| Grouping based on the optimal cut-off point of steroid decrease velocity |  | |  | |
| Slow | 1 | |  | |
| Medium | 0.65 (0.40-1.06) | | 0.083 | |
| Rapid | 0.46 (0.28-0.75) | | 0.002 | |
| **Treatment failure as defined by DFS** |  | |  | |
| Refined Minnesota aGVHD risk score before basiliximab treatment |  | |  | |
| Standard risk | 1 | |  | |
| High risk | 1.75 (1.07–2.85) | | 0.025 | |
| HCT-CI score^*^ |  | |  | |
| Low risk | 1 | |  | |
| Intermediate risk | 1.58 (1.01–2.50) | | 0.048 | |
| High risk | 3.72 (2.07–6.69) | | <0.001 | |
| Pretransplant chemotherapy |  | |  | |
| no | 1 | |  | |
| yes | 1.65 (1.01–2.69) | | 0.048 | |
| Grouping based on the optimal cut-off point of steroid decrease velocity |  | |  | |
| Slow | 1 | |  | |
| Medium | 0.73 (0.47-1.16) | | 0.183 | |
| Rapid | 0.48 (0.30-0.79) | | 0.003 | |
| **Non-relapse mortality** |  | |  | |
| Pretransplant chemotherapy |  | |  | |
| no | 1 | |  | |
| yes | 1.90 (1.16–3.10) | | 0.010 | |
| steroid dosage at the onset of treatment for acute GVHD | |  | |  |
| ≤ 1mg/kg | | 1 | |  |
| > 1mg/kg | | 2.97 (0.1.18-7.514) | | 0.021 |
| Grouping based on the optimal cut-off point of steroid decrease velocity | |  | |  |
| Slow | | 1 | |  |
| Medium | | 0.52 (0.26-1.07) | | 0.077 |
| Rapid | | 0.27 (0.10-0.76) | | 0.012 |

1. **Supplemental figures**


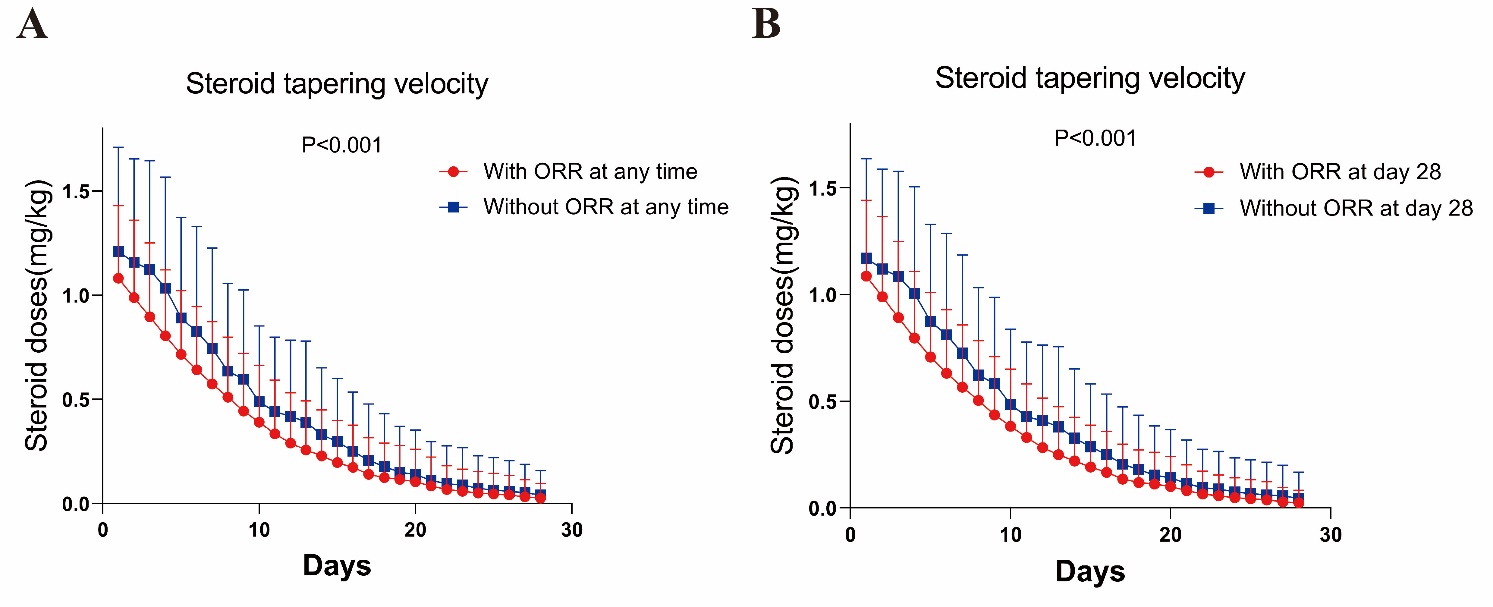


**Supplementary Figure 1** The steroid decreasing velocity curve within 28 days in patients with or without achieving ORR at any time (A) or at day 28 (B)


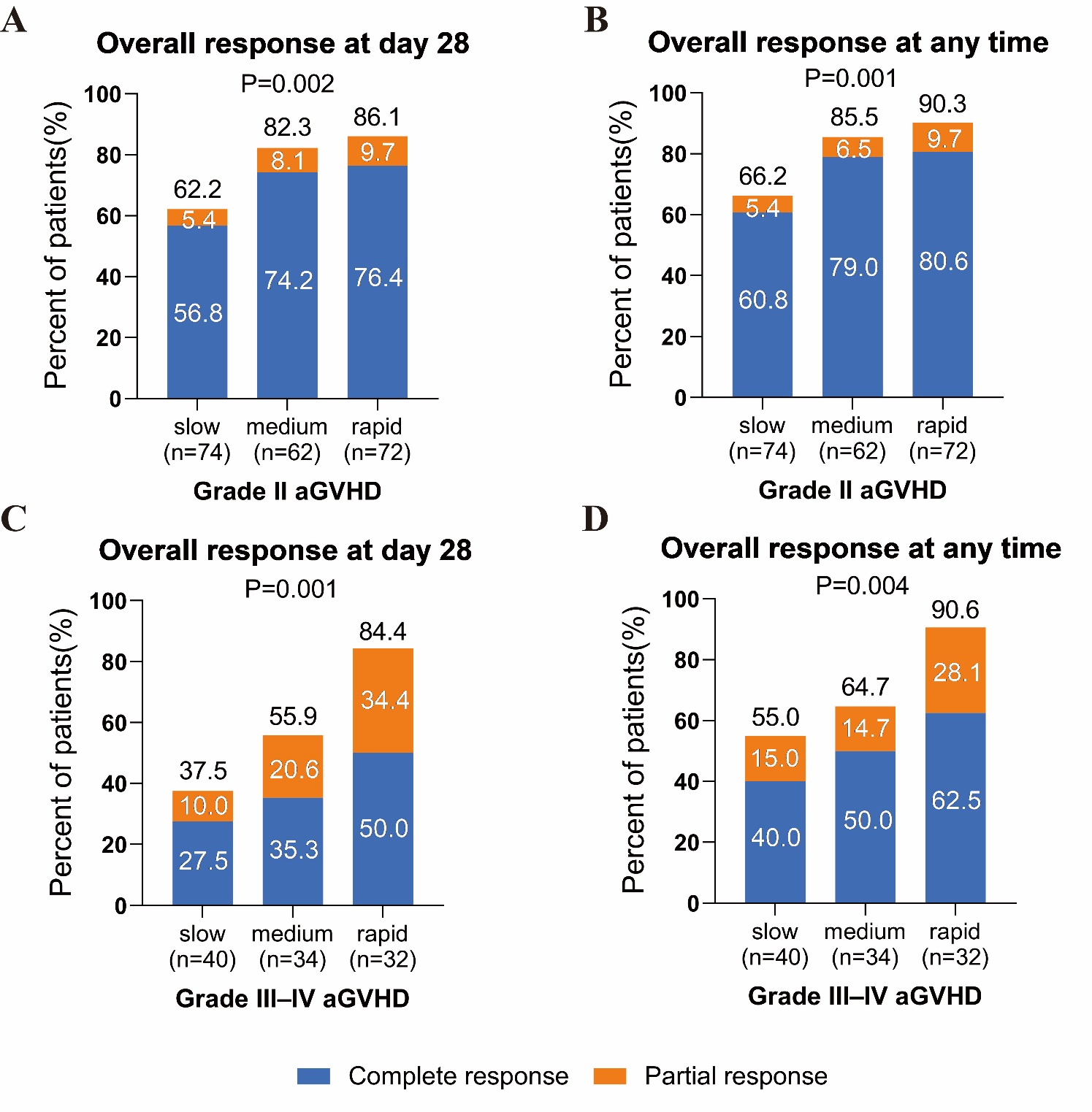


**Supplementary Figure 2** Overall response rate after basiliximab treatment between steroid decrease time subgroups of SR-aGVHD patients divided into grade II aGVHD patients and grade III-IV aGVHD patients according to refined Minnesota aGVHD risk score. The overall response rate (A) at day 28 or (B) at any time in grade Ⅱ aGVHD patients; (C) at day 28 or (D) at any time in grade Ⅲ-Ⅳ aGVHD patients


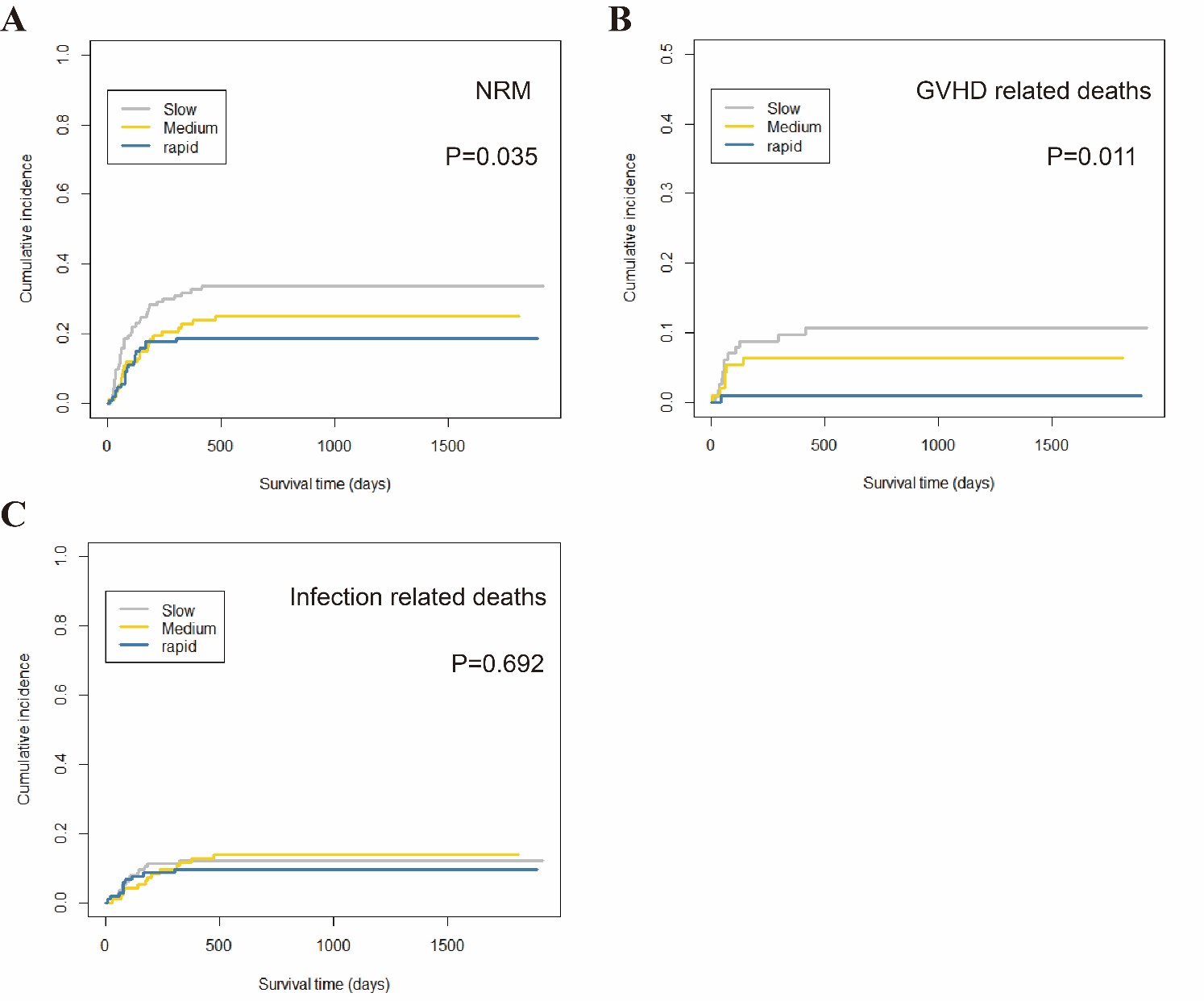


**Supplementary Figure 3** The cumulative incidence of NRM (A), GVHD related deaths (B) and infection related deaths (C) after 1 years of basiliximab treatment between steroid decrease time subgroups
